# NEIGHBORHOODS MATTER: ASSESSING THE IMPACT OF NEIGHBORHOOD FACILITIES ON SOCIAL ENGAGEMENT AND MENTAL WELL-BEING

**DOI:** 10.1093/geroni/igae098.2954

**Published:** 2024-12-31

**Authors:** Chengming Han, Nan Zhou

**Affiliations:** The University of Texas Medical Branch, Galveston, Texas, United States; Case Western Reserve University, Cleveland, Ohio, United States

## Abstract

Objective. This study investigated the effects of social engagement and neighborhood facilities on depressive symptoms (CESD) levels among older adults. Methods. Data were draw from the China Health and Retirement Longitudinal Study (CHARLS) 2011-2020. The analytical sample included 3,930 respondents who are 65 years older. An instrumental regression via the Generalized Method of Moments (GMM) was employed to estimate the social engagement and CESD scores at baseline, with the number of neighborhood facilities and function limitations (poverid=1.000, pendogen=0.000) serving as instrumental variables. A crossed random multilevel regression clustered within individuals and neighborhoods was used to examine the effects of neighborhood facilities, functional limitations, and social engagement on CESD scores. Results. The instrumental regression indicated a significant negative relationship between social engagement and CESD scores (b=-16.90, p < 0.000). Neighborhood facilities and functional limitations displayed significant effects on social engagement. The crossed random mixed model confirmed the negative association between social engagement (b= -0.24, p = 0.008) and CESD scores and highlighted the significant role of neighborhood facilities (b= -0.11, p < 0.000). The effects clustered at the neighborhood level (ICC=0.37) exhibit a greater impact compared to those clustered by individual ID (ICC=0.19) over survey periods. Conclusion. The findings of this study suggested that neighborhood-level factors have a more pronounced impact on social engagement and mental health than individual-level variations. These results highlighted the need for policies and interventions that enhance community facilities and promote social engagement to support the mental well-being of aging populations.

